# The Inflammatory Microenvironment in Colorectal Neoplasia

**DOI:** 10.1371/journal.pone.0015366

**Published:** 2011-01-07

**Authors:** Mairi H. McLean, Graeme I. Murray, Keith N. Stewart, Gillian Norrie, Claus Mayer, Georgina L. Hold, John Thomson, Nicky Fyfe, Mairi Hope, N. Ashley G. Mowat, Janice E. Drew, Emad M. El-Omar

**Affiliations:** 1 Gastrointestinal Research Group, School of Medicine and Dentistry, Aberdeen University, Aberdeen, United Kingdom; 2 Department of Pathology, Aberdeen University, Aberdeen, United Kingdom; 3 Colorectal Surgery Unit, Western General Hospital, Edinburgh, United Kingdom; 4 Biomathematics and Statistics Scotland, The Rowett Institute of Nutrition and Health, Aberdeen University, Aberdeen, United Kingdom; 5 The Rowett Institute of Nutrition and Health, Aberdeen University, Aberdeen, United Kingdom; Vanderbilt University Medical Center, United States of America

## Abstract

Colorectal cancer (CRC) is a major cause of mortality and morbidity worldwide. Inflammatory activity within the stroma of invasive colorectal tumours is known to be a key predictor of disease activity with type, density and location of immune cells impacting on patient prognosis. To date, there has been no report of inflammatory phenotype within pre-malignant human colonic adenomas. Assessing the stromal microenvironment and particularly, inflammatory activity within colorectal neoplastic lesions is central to understanding early colorectal carcinogenesis. Inflammatory cell infiltrate was assessed by immunohistochemistry in paired colonic adenoma and adjacent normal colonic mucosa samples, and adenomas exhibiting increasing degrees of epithelial cell dysplasia. Macrophage phenotype was assessed using double stain immunohistochemistry incorporating expression of an intracellular enzyme of function. A targeted array of inflammatory cytokine and receptor genes, validated by RT-PCR, was used to assess inflammatory gene expression. Inflammatory cell infiltrates are a key feature of sporadic adenomatous colonic polyps with increased macrophage, neutrophil and T cell (specifically helper and activated subsets) infiltration in adenomatous colonic polyps, that increases in association with characteristics of high malignant potential, namely, increasing degree of cell dysplasia and adenoma size. Macrophages within adenomas express iNOS, suggestive of a pro-inflammatory phenotype. Several inflammatory cytokine genes (*CXCL1*, *CXCL2*, *CXCL3*, *CCL20*, *IL8*, *CCL23*, *CCL19*, *CCL21*, *CCL5*) are dysregulated in adenomas. This study has provided evidence of increased inflammation within pre-malignant colonic adenomas. This may allow potential mechanistic pathways in the initiation and promotion of early colorectal carcinogenesis to be identified.

## Introduction

Colorectal cancer (CRC) is a major health burden causing significant morbidity and mortality, with over a million cases diagnosed each year worldwide [1]. The majority of CRC is sporadic, that is, associated with no identifiable heritable genetic mutation. The accepted pathogenetic framework for this malignancy is the adenoma-carcinoma sequence [2]. In understanding pathogenesis of malignancy, the emphasis has previously rested on epithelial cell behaviour, but over the last decade, the focus has moved to the microenvironment as a whole and the importance of stromal cell and epithelial cell interaction is now widely recognised [3], [4]. Inflammatory activity within the stroma of invasive colorectal tumours is known to be a key predictor of disease activity with type, density and location of immune cells impacting on patient prognosis [5]. The point at which this inflammatory infiltrate exerts influence on colorectal carcinogenesis is unknown. In breast cancer models, non-invasive tumour cells have been shown to recruit macrophages which induce angiogenesis and promote malignant transformation [6]. To date, there has been no report of inflammatory phenotype within pre-malignant human colonic adenomas. Assessing the stromal microenvironment and particularly, inflammatory activity within colorectal neoplastic lesions is central to understanding early colorectal carcinogenesis, and in particular, understanding the mechanisms involved in the transition of pre-invasive to invasive disease. The aim of this paper therefore was to define the inflammatory microenvironment within pre-malignant human adenomatous colonic polyps, and to investigate how this changes along with adenoma characteristics linked to high malignant potential.

## Materials and Methods

### Assessment of inflammatory cell phenotype

Inflammatory cell phenotype was assessed by immunohistochemistry on 65 colonic adenomatous polyps and 36 adjacent normal mucosal biopsies, obtained from 36 patients at CRC screening colonoscopy, as described previously [7]. The polyp sample set was expanded to include 40 colonic adenomas exhibiting low grade dysplasia (LGD), 40 with high grade dysplasia (HGD) and 40 adenomatous polyps with supervening invasive adenocarcinoma (cancer polyps (CaP)) [7]. Each specimen was evaluated to confirm histological diagnosis by an experienced consultant gastrointestinal pathologist (GIM). The macroscopic size of each polyp was measured post fixation, providing a consistent measurement of size for all samples. Within the prospectively collected polyp set, 36 were small (≤1cm) and 29 large (>1cm). There was no statistically significant difference in size distribution between the 40 LGD polyps, 40 HGD polyps or the 40 CaP (LGD vs. HGD, p = 0.548; HGD vs. CaP, p = 0.397; LGD vs. CaP, p = 0.068). Tissue was stained using the Envision+™ biotin free system [8] incorporating either the Envision+™ peroxidase linked biotin free system (Dako, K5007) or CSA II biotin-free tyramide signal amplification (Dako, K1497), dependant on antibody requirements (Table 1). Primary antibody was applied for 60 minutes following antigen retrieval (Table 1). Colour was developed with diaminobenzidine (DAB). One microscopic high powered field (HPF) from each sample was digitally imaged (×40 magnification) by a gastrointestinal pathologist (GIM) as representative of tissue type, distinct from lymphoid aggregation and within the area of most positive staining. The number of positive cells was counted to give a score of inflammatory cellular infiltrate.

**Table 1:** Characteristics of the antibodies used for the immunohistochemical analysis of inflammatory cell infiltrate.

Inflammatory cell type	Inflammatory cell marker	Type	Antigen retrieval method*	Positive tissue control	Dilution	IHC detection protocol	Supplier	Code	Isotype/Clone
T helper cell	CD4	mouse monoclonal	M20-high pH	Tonsil	1∶100	Envision	Novocastra	NCL-CD4-IF6	IgG_1_, IF6
cytotoxic T cell	CD8	mouse monoclonal	M20	Tonsil	1∶160	Envision	Dako	M7103	IgG_1κ_, C8/144B
activated T cell	CD25	mouse monoclonal	M20	Tonsil	1∶600	Tyramide signal amplification	Novocastra	NCL-CD25-305	IgG_2b_, 4C9
B cell	CD20	mouse monoclonal	M20	Tonsil	1∶400	Envision	Dako	M0755	IgG_2aκ_, L26
Plasma cell	CD138	mouse monoclonal	M20	Tonsil	1∶1000	Envision	Dako	M7077	IgG_1κ_, VS38C
NK cell	CD56	mouse monoclonal	M20	Appendix	1∶150	Envision	Monosan	MON9006-1	IgG_1_
Macrophage	CD68	mouse monoclonal	T16/M20	Tonsil	1∶300	Envision	Dako	M0814	IgG_1κ_, KP1[4]
Classically activated macrophage	iNOS	mouse monoclonal	M20	Appendix/colorectal carcinoma	1∶400	Tyramide signal amplification	BD biosciences	610328	6, IgG2a
Alternatively activated macrophage	Arginase I	rabbit polyclonal	M20	Liver	1∶40	Envision	Santa Cruz	sc-20150	H-52
Neutrophil	Neutrophil elastase	mouse monoclonal	nil	Tonsil	1∶100	Envision	Dako	M0752	IgG_1κ_, NP57
Mast cell	Mast cell tryptase	mouse monoclonal	M20	Tonsil/Appendix	1∶30,000	Envision	Dako	M7052	IgG_1κ_, AA1

*M = heat induced by microwaving in 10mM citrate buffer, pH 6.0., T = enzymatic digestion performed at 37°C, in 0.2% trypsin, 0.1% calcium chloride solution, pH 7.8., numerical values = time for antigen retrieval.

### Assessment of macrophage phenotype

Macrophage phenotype was assessed in 42 adenomatous polyps and 25 adjacent normal mucosal biopsies (randomly selected from the larger prospectively collected sample set) and polyps of increasing epithelial cell dysplasia, as described above. A double stain immunohistochemical technique was used, incorporating detection of an intracellular enzymatic marker of macrophage function, namely iNOS (pro-inflammatory classically activated macrophage) or arginase I (alternatively activated macrophage) [9], [10](Table 1), identified by peroxidase linked immunoreactivity. Sequential staining with CD68 detected macrophage infiltrate using an alkaline phosphatase linked detection signal. Staining was performed using either the G2 doublestain Envision™ kit or Envision™ doublestain kit, dependant on supplier availability (Dako, K5361 & K1395, respectively). Heat induced antigen retrieval was performed. Levamisole was added to the liquid permanent red chromagen to suppress endogenous alkaline phosphatase activity. Optimisation ensured no cross-reactivity or quenching of signal. The area of most positive macrophage infiltration within one HPF (×40 magnification), distinct from lymphoid aggregation, was identified under fluorescent light at 580 nm using a Texas red filter set and digitally imaged, and also captured under standard optical light. In relation to degree of cell dysplasia, the area of defined histological abnormality was marked and digitally imaged. The 2 images were imported into Corel-Paint X3, version 13 and macrophage infiltrate assessed for expression of either iNOS or arginase I.

### Profiling inflammatory gene expression

Tissue representative of each stage of the adenoma-carcinoma sequence (normal mucosa, adenoma and adenocarcinoma) was obtained from 7 colectomy specimens as part of an ongoing CRC tissue specimen bank, as previously published [11]. Histological analysis confirmed pathological diagnosis. RNA was extracted using the RNeasy mini kit (Qiagen, Crawley, UK, 74104), incorporating Qiashredder column tissue homogenisation and on-column DNase digestion. Agilent BioAnalyzer® profiling assessed yield and quality of the extracted total RNA. 500ng of total RNA was used to synthesize biotinylated cRNA using the Oligo-GEArray Reagent kit (Superarray Biosciences, USA, GA-034). Following quality assessment by Nanodrop spectrophotometry®, this was hybridised to a commercially available targeted Oligo-GEArray® gene filter, Human Inflammatory Cytokines and Receptors (OHS-011, Superarray Bioscience, USA), representing 112 inflammatory cytokine and receptor genes. Array images were captured using a Fuji LAS1000 cooled CCD camera. Hybridised arrays from each patient were imaged together. Signal intensity was analysed using AIDA Image Analyser programme v3.21 (Raytest Isotopenmessgeräte GmBH, Straubenhardt, Germany). Microarray data was validated by quantitative reverse transcriptase real-time PCR. Reverse transcription was performed using 500ng total RNA and random primers, catalyzed with Superscript™ II reverse transcriptase (concentration 200units/µl) (Invitrogen, UK, 18064-14). Glyceraldehyde-3-phosphate dehydrogenase (*GAPDH*) and beta-2-microglobulin (*B2M*) were selected as normaliser genes from the initial microarray filter gene expression data. All primers were obtained from Superarray Bioscience (UK distributer; Tebu-Bio, Peterborough, UK). Real-time PCR reaction for each sample was performed on a Biorad iCycler® (Bio-Rad Laboratories, UK) in duplicate, incorporating RT^2^ Real-Time™ SYBR green mastermix (PA-010)(Superarray Bioscience, USA). A 10 fold serial dilution standard curve was included for each gene of interest and both normalisers on each plate. Data was included if the PCR efficiency fell between 80% to 105%. A no template control was included. The PCR cycling programme (15 minutes, 95°C to activate the *Taq* polymerase, followed by 40 cycles of 95°C for 15 seconds, 55°C for 30 seconds and 72°C for 30 seconds) was followed immediately by a default melting curve program to 96°C. Sequence verification of PCR amplicons was performed by either cloning into pGEM®- T easy vector system (Promega, Southhampton, UK, A1360), incorporating transformation of JM109 high efficiency competent cells (Promega, Southhampton, UK, L2001) and the universal M13 primer, or purification of RT-PCR product and sequencing using custom designed gene specific primers based on reference positions indicated from RefSeq accession number supplied with Superarray primer assays.

### Statistical methods

Differences in inflammatory cell infiltrate between tissue types and in relation to adenoma size was assessed using paired t-tests. One way ANOVA assessed the relationship between inflammatory infiltrate and degree of dysplasia. One paired T test assessed macrophage phenotype. Both absolute and relative differences in function were tested in a one way ANOVA. SAS 9.1.3 for Windows XP (SAS Institute, Cary, NC, USA) was used for statistical analyses.

Gene array signal intensities were normalised to background signal, log-transformed and rescaled to ensure each data point lay between lowest and highest signal intensity. Data from short and long exposure images were analysed in combination and a weighted average generated. ANOVA was conducted with Patient as blocking variable. Gene expression signals between normal, adenoma and CRC were compared and greater than 2 fold difference in expression pattern identified. RT-PCR data was analysed using a paired t-test, incorporating the 2^−ΔΔ^ C_T_ (Livak) method as previously published [12].

Statistical significance was set at *p*<0.05 throughout.

### Ethics

Ethical approval for this study was obtained from Grampian Ethics Committee. Written informed consent was obtained from all participants.

## Results

### Assessment of inflammatory cell phenotype

**Figure 1:**
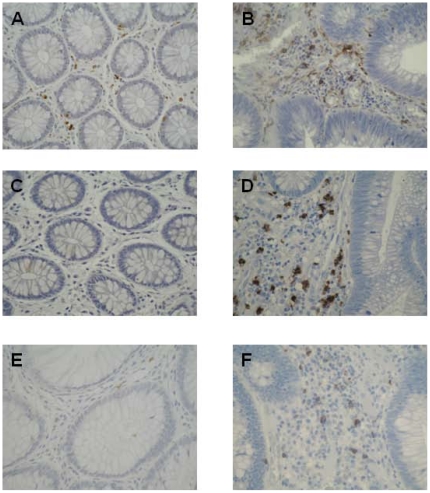
Inflammatory cell infiltration in colorectal adenomas compared to normal colonic mucosa. Macrophage infiltrate was increased in polyps (B) compared to adjacent normal mucosa (A). Neutrophil infiltrate was increased in polyps (D) compared to adjacent normal mucosa (C). CD25^+^ activated T cells were increased in polyps (F) compared to adjacent normal mucosa (E).

Patient and adenoma characteristics are shown in Table 2. Macrophage (p = 0.0002), neutrophil (p = 0.0001), helper T cells (p = 0.004),activated T cells (p = 0.0001) and NK cells (p = 0.04) were increased in adenomas compared to adjacent normal mucosa (Table 3 and Figure 1). Infiltration of macrophage, neutrophil and activated T cells correlated with adenoma size, with correlation co-efficient of 0.51 (p = 0.0001), 0.27 (p = 0.03) and 0.50 (p = 0.0001), respectively. This represented a mean cell count of 9 (95% CI 6–12) in small polyps compared to 12 (95% CI 6–19) in large polyps for neutrophil infiltrate (p = 0.0006), 12 (95% CI 9–14) in small polyps compared to 20 (95% CI 16–25) in large polyps for macrophage infiltrate (p = 0.003) and 2 (95% CI 1–3) in small polyps compared to 8 (95% CI 6–11) in large polyps for activated T cell infiltrate (p = 0.0001). T helper cells did not increase along with adenoma size (p = 0.23). There was an increase in macrophage (p = 0.0001) and neutrophils (p = 0.0001) as the degree of dysplasia progressed from low grade to high grade and finally to overt invasive adenocarcinoma (Table 4). There was a statistically significant increase in T helper cells in cancer polyps compared to their benign adenomatous counterparts (p = 0.009). There was no increase in activated T cell infiltration in association with increasing degree of cell dysplasia (p = 0.06).

**Table 2:** Adenoma and patient characteristics.

Adenoma and patient characteristics		Adenomas collected within paired polyp-normal sample set (n = 64)	LGD (n = 40)	HGD (n = 40)	CaP (n = 40)
**Colonic site**	Left sided*	54 (85%)	35 (88%)	37 (92%)	37 (92%)
	Right sided	10 (15%)	5 (12%)	3 (8%)	3 (8%)
**Polyp size**	Small (≤1cm)	35 (55%)	11 (27%)	8 (20%)	5 (12%)
	Large (>1cm)	29 (45%)	29 (73%)	32 (80%)	35 (88%)
**Histological classification**	Tubular adenoma	50 (78%)	32 (80%)	15 (38%)	13 (32%)
	Tubulo-villous adenoma	14 (22%)	8 (20%)	25 (62%)	27 (68%)
**Degree of dysplasia**	Low grade	56 (88%)	0 (0%)	0 (0%)	0 (0%)
	High grade	2 (3%)	40 (100%)	0 (0%)	0 (0%)
	Cancer polyp	6 (9%)	0 (0%)	40 (100%)	40 (100%)
**Source**	CRC screening	64 (100%)	32 (80%)	2 (5%)	5 (12%)
	Diagnostic database	0 (0%)	8 (20%)	38 (95%)	35 (88%)
**Patient age (years)**		63 (53–69)	63 (55–79)	71 (45–92)	67 (38–89)
**Gender**	Male	58%	60%	55%	53%
	Female	42%	40%	45%	47%

*distal to splenic flexure.

**Table 3:** Assessment of inflammatory cell phenotype in 65 colonic adenomas and 35 adjacent normal mucosal biopsies (paired T test).

Inflammatory cell	Normal Colon	Adenomatous Polyp	Mean Difference (95% CI)	P-Value
T helper cell	13.19 (11.84)	21.80 (14.67)	8.61 (2.95, 14.27)	**0.004**
Cytotoxic T cell	6.25 (7.97)	6.00 (8.31)	−0.25 (−2.66, 2.16)	0.83
B cell	2.78 (6.56)	4.11 (7.31)	1.33 (−1.60, 4.27)	0.36
Activated T cell	2.32 (2.67)	7.69 (5.71)	5.53 (3.34, 7.72)	**<0.0001**
NK cell	0.19 (1.01)	2.11 (5.39)	1.92 (0.11, 3.72)	**0.04**
Macrophage	10.83 (10.20)	19.63 (10.69)	8.91 (4.50, 13.32)	**0.0002**
Mast cell	10.00 (5.35)	10.56 (9.63)	0.71 (−1.97, 3.40)	0.59
Neutrophil	1.56 (3.22)	14.83 (14.76)	13.28 (8.37, 18.19)	**<0.0001**
Plasma cell	5.50 (7.19)	6.00 (8.18)	0.50 (−2.61, 3.61)	0.75

In this analysis, the maximum infiltrate value was used for the polyp when more than 1 polyp existed.

Numerical data represents mean cell counts (with mean of standard deviation).

**Table 4:** Assessment of inflammatory cell infiltrate in colonic adenomas in relation to increasing degree of epithelial cell dysplasia (one way ANOVA).

Inflammatory cell	Low grade dysplasia	High grade dysplasia	Cancer polyps	P value
T helper cell	14.20 (40)	13.80 (40)	21.95 (38)	**0.009**
Activated T cell	4.95 (40)	5.43 (40)	8.26 (39)	0.06
Macrophage	40.37 (39)	55.89 (40)	82.33 (39)	**<0.0001**
Neutrophil	8.13 (40)	17.80 (40)	31.44 (39)	**<0.0001**

Numerical data denotes mean cell counts. () = n.

### Assessment of macrophage phenotype

There were no iNOS positive pro-inflammatory macrophages in the normal colonic mucosal biopsies. In comparison, within paired adenomas, these cells were a key feature of the stroma with 84% (61%–93%) of the macrophage population expressing iNOS (p = 0.001) (Table 5). Arginase I expression within the macrophage population was not a prominent feature of either the normal mucosal biopsies or the adenomas. Overall, there appeared to be more arginase I expressing macrophages within the stroma of the polyps, when analysed as both the absolute number of arginase I positive cells and the percentage of arginase I positive cells within the total macrophage population, and this was statistically significant with p = 0.001 and p = 0.023, respectively, but the overall number of positive cells were low (Table 5). 45%, 67% and 34% of the total macrophage population expressed iNOS in the low grade dysplastic, high grade dysplastic and cancer polyp groups, respectively. This is compared to 12%, 5% and 13% of macrophage expressing arginase I. The difference in this pattern of enzymatic expression favours a pro-inflammatory phenotype. The relative proportion of iNOS expressing pro-inflammatory cells to arginase I expressing regulatory cells was greatest in the low grade dysplastic (p = 0.001) and high grade dysplastic groups (p = 0.001). The relative proportion of regulatory to pro-inflammatory macrophage was higher in the cancer polyp group suggesting that regulatory macrophage are more abundant within areas of invasive disease.

**Table 5:** Assessment of macrophage phenotype in colonic adenomas (n = 42) and adjacent normal mucosa (n = 25). () = 25^th^ and 75^th^ centiles.

	Normal colon	Adenomatous polyp	P value
Median number of iNOS^+^ cells	0 (0–3)	11 (6–18)	0.001
% iNOS^+^ cells/total CD68^+^ cells	0% (0%–43%)	84% (61%–93%)	0.001
Median number of arginase I^+^ cells	0 (0–1)	1 (0–2)	0.001
% arginase I^+^ cells/total CD68^+^ cells	0% (0%–10%)	6% (0%–25%)	0.023

### Profiling of inflammatory gene expression

The average age of the patients was 71 years (range 55–79). Five of the patients were male. Five of the cancers originated on the left side of colon. On staging, one was Duke's A, 4 Dukes B and 2 Dukes C.

**Figure 2:**
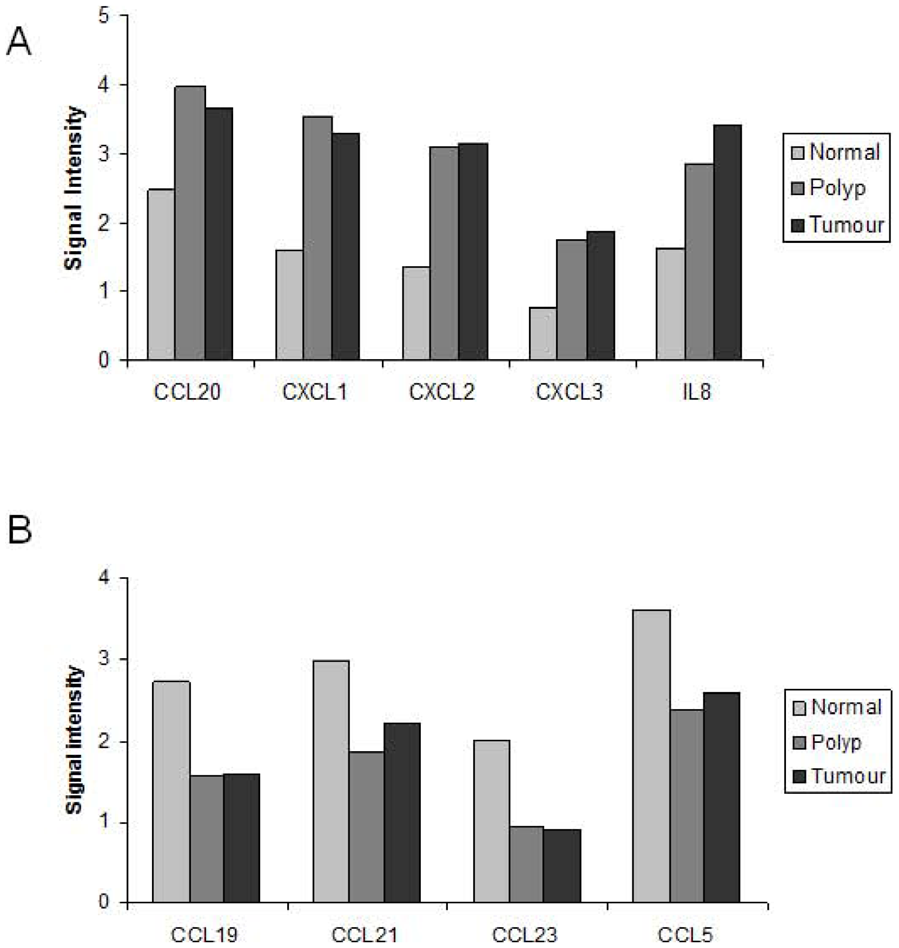
Signal intensity of targeted microarray. Signal intensity of targeted microarray with gene expression in 5 genes significantly up-regulated in adenoma and CRC compared to normal (A) and 4 genes significantly down-regulated in adenoma and CRC compared to adjacent normal mucosa (B) (p values noted in Table 6).

*CXCL1*, *CXCL2*, *CXCL3*, *CCL20*, and *IL-8* had increased expression in the adenoma and adenocarcinoma compared to normal colonic mucosa. *CCL19*, *CCL21*, *CCL23*, *CCL5*, were found to have reduced expression in the adenoma and adenocarcinoma compared to normal mucosa (Table 6, and Figure 2). It is clear that the change in expression of all of these genes occurs in the precancerous adenomatous lesion, early in the neoplastic process, prior to malignant transformation. Expression pattern of all of these genes was validated by RT-PCR, normalised to both housekeeper genes (*B2M* and *GAPDH*) (data for *GAPDH* shown in Table S1). Identification of the PCR products was confirmed by sequencing of the PCR amplicons.

**Table 6:** Differential expression of inflammatory genes in colorectal neoplastic progression.

Gene	Normal(N)	Adenoma(Ad)	CRC	P value N.vs.Ad	P value N.vs.CRC	P value Ad.vs.CRC
**CCL20**	2.47	3.97	3.65	0.014	0.044	0.537
**CXCL1**	1.60	3.52	3.31	0.001	0.001	0.520
**CXCL2**	1.35	3.10	3.16	0.003	0.002	0.910
**CXCL3**	0.77	1.76	1.88	0.009	0.004	0.712
**IL-8**	1.63	2.84	3.42	0.01	0.001	0.173
*CCL5*	3.61	2.40	2.59	0.007	0.017	0.660
*CCL19*	2.73	1.57	1.59	0.004	0.004	0.944
*CCL21*	2.98	1.87	2.23	0.007	0.052	0.285
*CCL23*	2.02	0.96	0.91	0.001	0.001	0.831

Gene expression in normal colonic mucosa vs. pre-malignant adenomatous polyp (Ad) vs. invasive adenocarcinoma (CRC). Bold denotes genes with increased expression in neoplastic tissue compared to normal mucosa, and italics denotes genes with reduced expression in neoplastic tissue compared to normal mucosa.

## Discussion

This study has defined the inflammatory cell phenotype within the stromal microenvironment of human colorectal pre-malignant adenomas and identified how this evolves with adenoma progression to overt invasive disease. In addition, several pro-inflammatory genes are differentially expressed in pre-malignant colonic adenomatous polyp compared to adjacent normal mucosa. All of the 9 differentially expressed genes are linked to inflammation and key cell functions important in tumour biology. IL-8 is a known potent chemoattractant of neutrophils and macrophages to areas of inflammatory activity. IL-8 is also mitogenic [13], angiogenic [14] and, through linked expression of MMP's, influences tumour cell motility such that invasion is enhanced [15]. CCL20 is a chemokine involved in directing lymphoid cell migration through binding to its receptor CCR6. CCR6 is significantly up-regulated in both colorectal cancer and associated liver metastases and data suggests that it plays a role in the recruitment of CCR6^+^ tumour cells to the site of distant metastases [16]. CXCL1 expression is up-regulated in colorectal adenomas and adenocarcinoma [17], inhibiting apoptosis and inversely linked to expression of fibulin-1, an extra-cellular matrix protein implicated in control of tumour cell migration. Wang *et al.* (2006) [18] reported that expression of CXCL1 induced by PGE_2_ was linked to angiogenesis in colorectal cancer *in vitro* and *in vivo* and thus provided a link between COX-2 up-regulation and chemokine induced tumour associated endothelial cell migration. Of interest, is the fact that expression of several chemokines was down-regulated in adenomas. CCL23 is an immune mediator involved in the chemotaxis of monocytes but not neutrophils [19]. CCL23 inhibits the release of neutrophils and monocytes from bone marrow, suggesting that it may be an important mediator in regulating bone marrow response during immune stimulation [20]. In addition, CCL23 confers angiogenic properties [21], mediated through up-regulation of MMP2 gene expression in endothelial cells [22]. CCL5 drives T cell and monocyte migration and activation, with increased expression linked to a number of malignancies [23], [24]. CCL5 was not found to be up-regulated in colorectal cancer compared to adjacent normal mucosa by Baier and colleagues [25] and this would be in keeping with the present study. CCL19 and CCL21 are structurally related chemokines that share the common receptor, CCR7. They play a pivotal role in the development of secondary lymphoid tissue as seen in animal models of deficiency [26], [27] and are involved in T cell activation through interaction with dendritic cells within secondary lymphoid organs. It is difficult to speculate on the biological ramifications of differential expression of each of these chemokines individually. However, the dysregulation of these inflammatory cytokine and chemokine genes in adenomatous polyps compared to adjacent normal mucosa complements the cellular findings of an active inflammatory stromal microenvironment and is worthy of further investigation.

Several gene expression studies have previously been performed on normal and diseased colonic tissue. In general, a genome wide approach has been applied to identify genes dysregulated in malignant tissue that can be targeted in treatment strategies and for staging [28]–[30]. In contrast, there are fewer reports of gene expression studies in pre-malignant colorectal adenomas [31]–[34]. Each stage of the adenoma-carcinoma sequence may have an identifying gene signature primarily involving genes related to cell cycle, cell growth, RNA and protein processing and cell signalling. Sabates-Bellver *et al* [35] assessed 32 pedunculated colorectal adenomas compared to normal mucosa and identified differential expression of several inflammatory genes in adenomas, corresponding to those identified from our data analysis. Specifically, *CCL19* and *CCL5* had reduced expression in polyp lesions. In addition, *IL-8*, *CXCL1*, *CXCL2*, *CXCL3*, and *CCL20* had higher levels of expression in the polyps compared to normal, which is in keeping with the current study.

The gut exerts a continuous low grade ‘physiological’ mucosal inflammatory activity, in response to continual challenges from luminal contents, with T helper cell, macrophage, CD8^+^ T cells, and plasma cell infiltrates [36], [37]. The normal mucosal biopsies in this study revealed mucosal immunoactivity as expected. The demonstration that polyps harbour an increased cellular inflammatory infiltrate over and above what is expected in “normal” colonic tissue is an important finding. This study has shown that adenomatous polyps are rich with pro-inflammatory macrophage, neutrophil and T helper cell infiltration and these are likely to exert a significant influence on their surrounding microenvironment within the polyp lesion. NK cells were also found to be increased in polyp compared to normal mucosa. However, the majority of polyps did not have any evidence of CD56^+^ NK cell infiltrate and those that did had an average of only 5 positive cells/HPF. This only marginally achieved statistical significance (p = 0.04) and the relevance of this in the biological microenvironment is debatable and requires further evaluation.

Neutrophils are a source of N- nitrosamines, especially in the presence of colonic amine producing bacteria, and these are known to be carcinogenic [38], [39]. Coussens *et al* [40] showed that neutrophil and macrophage derived MMP-9 enhanced progression from dysplasia to overt malignancy through paracrine signalling in a mouse model of squamous skin cancer, with reduced MMP-9 delaying angiogenesis within dysplastic areas and reducing the incidence of invasive disease. In addition, inflammatory cells of haemopoetic origin, introduced by bone marrow transplant, were identified in hyperplastic, dysplastic and neoplastic skin lesions, and produced MMP-9 driven changes associated with early carcinogenesis. Our previous data showed that MMP-9 is expressed within adenomatous polyps [41]. Recently, Jablonska and colleagues [42] have demonstrated that endogenous IFNβ inhibits angiogenesis, by inhibiting pro-angiogenic genes within tumour associated neutrophils, in a transplantable mouse tumour model. Neutrophil elastase, secreted by neutrophils, is a main constituent of phagocytic response, degrading proteins within the local environment such as elastin, collagen and other constituents of extracellular matrix [43]. Elastase deficient mice have significantly reduced skin carcinoma load in response to repeated UV light or chemical exposure [44]. Administration of a specific neutrophil elastase inhibitor suppressed the proliferation and motility of a pancreatic cancer cell line [45], as well as squamous and adenocarcinoma cell lines of lung malignancy [46]. The underlying mechanism is unclear. Degradation of protein transcription factors may disrupt regulatory proteins of gene expression [44]. Reactive oxygen species derived from polymorphs can indirectly affect gene expression by, for example, modulating enzyme activity and promoting changes in transcription factor binding capacity. Neutrophil activation has been implicated in cell cycle G2/M arrest, dependent on expression of p53 and p21, and associated with DNA damage checkpoint mechanisms, in an *in vitro* co-culture model mimicking colitis [47].

Pro-inflammatory macrophages are a central and potent constituent of innate immunity. Within our cancer polyp group, the macrophage population appears to change with a reduced proportion of iNOS expressing classically activated cells. Macrophages are well recognised at sites of malignancy. Tumour associated macrophage have poor antigen presenting capabilities with a limited anti-tumour response. This reduced tumoricidal capacity has been linked to reduced iNOS expression [48] and would be in keeping with the current data.

There is pharmacological evidence for the role of inflammation in the development of colorectal neoplasia. Several large randomised controlled trials have shown that regular aspirin use can reduce the risk of CRC and adenoma development by up to 50% [49]–[52]. The use of aspirin or NSAIDs in our study population is unknown.

This study has defined the stromal microenvironment of pre-malignant colorectal adenomas and identified key inflammatory components involved in adenoma progression to invasive malignancy. It is clear from our data that a phenotypic and genotypic ‘switch’ occurs early in the adenoma-carcinoma sequence, with expression of inflammatory cytokines and chemokines dysregulated in the transition from normal mucosa to adenomatous polyp, rather than at the polyp to invasive disease transition. This data increases the understanding of the environmental influences within the adenoma in relation to disease progression and may have identified potential mechanistic pathways in the initiation and promotion of early colorectal carcinogenesis. Ultimately, this may identify phenotypic markers within adenomas which determine malignant potential.

## Supporting Information

Table S1 RT-PCR validation of gene expression in normal colonic mucosa, adenomatous polyp and adenocarcinoma (CRC), normalised to expression of GAPDH and B2M. P value generated from 2 tailed t-test statistical analysis. Bold denotes genes with increased expression in neoplastic tissue compared to normal mucosa, and italics denotes genes with reduced expression in neoplastic tissue compared to normal mucosa.
(DOC)

## References

[1] Cancer Research UK Cancer statistics.. http://www.cancerresearchuk.org.

[2] Fearon ER, Vogelstein B (1990). A genetic model for colorectal tumorigenesis.. Cell.

[3] Mantovani A, Romero P, Palucka AK, Marincola FM (2008). Tumour immunity: effector response to tumour and role of the microenvironment.. Lancet.

[4] Lorusso G, Ruegg C (2008). The tumor microenvironment and its contribution to tumor evolution toward metastasis.. Histochem Cell Biol.

[5] Galon J, Costes A, Sanchez-Cabo F, Kirilovsky A, Mlecnik B (2006). Type, density, and location of immune cells within human colorectal tumors predict clinical outcome.. Science.

[6] DeNardo DG, Coussens LM (2007). Inflammation and breast cancer. Balancing immune response: crosstalk between adaptive and innate immune cells during breast cancer progression.. Breast Cancer Res.

[7] McLean MH, Murray GI, Fyfe N, Hold GL, Mowat NA (2008). COX-2 expression in sporadic colorectal adenomatous polyps is linked to adenoma characteristics.. Histopathology.

[8] Kumarakulasingham M, Rooney PH, Dundas SR, Telfer C, Melvin WT (2005). Cytochrome p450 profile of colorectal cancer: identification of markers of prognosis.. Clin Cancer Res.

[9] Munder M, Eichmann K, Moran JM, Centeno F, Soler G (1999). Th1/Th2-regulated expression of arginase isoforms in murine macrophages and dendritic cells.. J Immunol.

[10] Mosser DM (2003). The many faces of macrophage activation.. J Leukoc Biol.

[11] Duncan R, Carpenter B, Main LC, Telfer C, Murray GI (2008). Characterisation and protein expression profiling of annexins in colorectal cancer.. Br J Cancer.

[12] Livak KJ, Schmittgen TD (2001). Analysis of relative gene expression data using real-time quantitative PCR and the 2(-Delta Delta C(T)) method.. Methods.

[13] Ishiko T, Mita S, Hidaka H, Kamohara H, Ikeda O (2003). Human carcinoma cells express IL-8 and IL-8 receptor: their role and regulation in cancer biology.. International Congress Series.

[14] Kitadai Y, Takahashi Y, Haruma K, Naka K, Sumii K (1999). Transfection of interleukin-8 increases angiogenesis and tumorigenesis of human gastric carcinoma cells in nude mice.. Br J Cancer.

[15] Mian BM, Dinney CP, Bermejo CE, Sweeney P, Tellez C (2003). Fully human anti-interleukin 8 antibody inhibits tumor growth in orthotopic bladder cancer xenografts via down-regulation of matrix metalloproteases and nuclear factor-kappaB.. Clin Cancer Res.

[16] Rubie C, Oliveira V, Kempf K, Wagner M, Tilton B (2006). Involvement of chemokine receptor CCR6 in colorectal cancer metastasis.. Tumour Biol.

[17] Wen Y, Giardina SF, Hamming D, Greenman J, Zachariah E (2006). GROalpha is highly expressed in adenocarcinoma of the colon and down-regulates fibulin-1.. Clin Cancer Res.

[18] Wang D, Wang H, Brown J, Daikoku T, Ning W (2006). CXCL1 induced by prostaglandin E2 promotes angiogenesis in colorectal cancer.. J Exp Med.

[19] Forssmann U, Delgado MB, Uguccioni M, Loetscher P, Garotta G (1997). CKbeta8, a novel CC chemokine that predominantly acts on monocytes.. FEBS Lett.

[20] Shih CH, van Eeden SF, Goto Y, Hogg JC (2005). CCL23/myeloid progenitor inhibitory factor-1 inhibits production and release of polymorphonuclear leukocytes and monocytes from the bone marrow.. Exp Hematol.

[21] Hwang J, Son KN, Kim CW, Ko J, Na DS (2005). Human CC chemokine CCL23, a ligand for CCR1, induces endothelial cell migration and promotes angiogenesis.. Cytokine.

[22] Son KN, Hwang J, Kwon BS, Kim J (2006). Human CC chemokine CCL23 enhances expression of matrix metalloproteinase-2 and invasion of vascular endothelial cells.. Biochem Biophys Res Commun.

[23] Vaday GG, Peehl DM, Kadam PA, Lawrence DM (2006). Expression of CCL5 (RANTES) and CCR5 in prostate cancer.. Prostate.

[24] Sutton A, Friand V, Papy-Garcia D, Dagouassat M, Martin L (2007). Glycosaminoglycans and their synthetic mimetics inhibit RANTES-induced migration and invasion of human hepatoma cells.. Mol Cancer Ther.

[25] Baier PK, Eggstein S, Wolff-Vorbeck G, Baumgartner U, Hopt UT (2005). Chemokines in human colorectal carcinoma.. Anticancer Res.

[26] Gunn MD, Kyuwa S, Tam C, Kakiuchi T, Matsuzawa A (1999). Mice lacking expression of secondary lymphoid organ chemokine have defects in lymphocyte homing and dendritic cell localization.. J Exp Med.

[27] Mori S, Nakano H, Aritomi K, Wang CR, Gunn MD (2001). Mice lacking expression of the chemokines CCL21-ser and CCL19 (plt mice) demonstrate delayed but enhanced T cell immune responses.. J Exp Med.

[28] Ichikawa Y, Ishikawa T, Takahashi S, Hamaguchi Y, Morita T (2002). Identification of genes regulating colorectal carcinogenesis by using the algorithm for diagnosing malignant state method.. Biochem Biophys Res Commun.

[29] Tang ZQ, Han LY, Lin HH, Cui J, Jia J (2007). Derivation of stable microarray cancer-differentiating signatures using consensus scoring of multiple random sampling and gene-ranking consistency evaluation.. Cancer Res.

[30] Daemen A, Gevaert O, De Bie T, Debucquoy A, Machiels JP (2008). Integrating microarray and proteomics data to predict the response on cetuximab in patients with rectal cancer.. Pac Symp Biocomput.

[31] Notterman DA, Alon U, Sierk AJ, Levine AJ (2001). Transcriptional gene expression profiles of colorectal adenoma, adenocarcinoma, and normal tissue examined by oligonucleotide arrays.. Cancer Res.

[32] Lin YM, Furukawa Y, Tsunoda T, Yue CT, Yang KC (2002). Molecular diagnosis of colorectal tumors by expression profiles of 50 genes expressed differentially in adenomas and carcinomas.. Oncogene.

[33] Kita H, Hikichi Y, Hikami K, Tsuneyama K, Cui ZG (2006). Differential gene expression between flat adenoma and normal mucosa in the colon in a microarray analysis.. J Gastroenterol.

[34] Sillars-Hardebol AH, Carvalho B, de Wit M (2010). Identification of key genes for carcinogenic pathways associated with colorectal adenoma-to-carcinoma progression.. Tumour Biol.

[35] Sabates-Bellver J, Van der Flier LG, de Palo M, Cattaneo E, Maake C (2007). Transcriptome profile of human colorectal adenomas.. Mol Cancer Res.

[36] MacDonald TT (2003). The mucosal immune system.. Parasite immunology.

[37] Wittig BM, Zeitz M (2003). The Gut as an organ of immunology.. International Journal of Colorectal Disease.

[38] Grisham MB, Ware K, Gilleland HE, Gilleland LB, Abell CL (1992). Neutrophil-mediated nitrosamine formation: role of nitric oxide in rats.. Gastroenterology.

[39] Vermeer IT, Henderson LY, Moonen EJ, Engels LG, Dallinga JW (2004). Neutrophil-mediated formation of carcinogenic N-nitroso compounds in an in vitro model for intestinal inflammation ..

[40] Coussens LM, Tinkle CL, Hanahan D, Werb Z (2000). MMP-9 supplied by bone marrow-derived cells contributes to skin carcinogenesis.. Cell.

[41] Jeffery N, McLean MH, El-Omar EM, Murray GI (2009). The matrix metalloproteinase/tissue inhibitor of matrix metalloproteinase profile in colorectal polyp cancers.. Histopathology.

[42] Jablonska J, Leschner S, Westphal K, Lienenklaus S, Weiss S (2010). Neutrophils responsive to endogenous IFN-beta regulate tumor angiogenesis and growth in a mouse tumor model.. J Clin Invest.

[43] Sun Z, Yang P (2004). Role of imbalance between neutrophil elastase and alpha 1-antitrypsin in cancer development and progression.. Lancet Oncol.

[44] Starcher B, O'Neal P, Granstein RD, Beissert S (1996). Inhibition of neutrophil elastase suppresses the development of skin tumors in hairless mice.. J Invest Dermatol.

[45] Kamohara H, Sakamoto K, Mita S, An XY, Ogawa M (1997). Neutrophil elastase inhibitor (ONO-5046.Na) suppresses the proliferation, motility and chemotaxis of a pancreatic carcinoma cell line, Capan-1.. Res Commun Mol Pathol Pharmacol.

[46] Inada M, Yamashita J, Ogawa M (1997). Neutrophil elastase inhibitor (ONO-5046-Na) inhibits the growth of human lung cancer cell lines transplanted into severe combined immunodeficiency (scid) mice.. Res Commun Mol Pathol Pharmacol.

[47] Campregher C, Luciani MG, Gasche C (2008). Activated neutrophils induce an hMSH2-dependent G2/M checkpoint arrest and replication errors at a (CA)13-repeat in colon epithelial cells.. Gut.

[48] Dinapoli MR, Calderon CL, Lopez DM (1996). The altered tumoricidal capacity of macrophages isolated from tumor-bearing mice is related to reduce expression of the inducible nitric oxide synthase gene.. J Exp Med.

[49] Thun MJ, Namboodiri MM, Heath CW (1991). Aspirin use and reduced risk of fatal colon cancer.. N Engl J Med.

[50] Baron JA, Cole BF, Sandler RS, Haile RW, Ahnen D (2003). A randomized trial of aspirin to prevent colorectal adenomas.. N Engl J Med.

[51] Sandler RS, Halabi S, Baron JA, Budinger S, Paskett E (2003). A randomized trial of aspirin to prevent colorectal adenomas in patients with previous colorectal cancer.. N Engl J Med.

[52] Benamouzig R, Deyra J, Martin A, Girard B, Jullian E (2003). Daily soluble aspirin and prevention of colorectal adenoma recurrence: one-year results of the APACC trial.. Gastroenterology.

