# Supplementary material for: The Inflammatory Microenvironment in Colorectal Neoplasia
Source: PLoS One. 2011 Jan 7;6(1):e15366. doi: 10.1371/journal.pone.0015366 (PMC3017541; doi:10.1371/journal.pone.0015366)
Supplement: Table S1 — RT-PCR validation of gene expression in normal colonic mucosa, adenomatous polyp and adenocarcinoma (CRC), normalised to expression of GAPDH and B2M. P value generated from 2 tailed t-test statistical analysis. Bold denotes genes with increased expression in neoplastic tissue compared to normal mucosa, and italics denotes genes with reduced expression in neoplastic tissue compared to normal mucosa. (DOC) [file pone.0015366.s001.doc]

| **Gene, normalised to GAPDH** | **Fold change in expression**  **normal vs. adenoma** | **Fold change in expression**  **normal vs. CRC** |
| --- | --- | --- |
| **CCL20** | 3.79 (p=0.048) | 3.88 (p=0.015) |
| **CXCL1** | 12.79 (p=0.001) | 16.35 (p=0.001) |
| **CXCL2** | 4.44 (p=0.029) | 9.13 (p=0.003) |
| **CXCL3** | 24.95 (p=0.001) | 34.78 (p=0.002) |
| **IL8** | 16.19 (p=0.003) | 50.09 (p=0.001) |
| *CCL5* | 0.17 (p=0.024) | 0.19 (p=0.010) |
| *CCL19* | 0.08 (p=0.006) | 0.11 (p=0.048) |
| *CCL21* | 0.02 (p=0.024) | 0.09 (p=0.007) |
| *CCL23* | 0.03 (p=0.001) | 0.06 (p=0.001) |

| **Gene, normalised to B2M** | **Fold change in expression**  **normal vs. adenoma** | **Fold change in expression**  **normal vs. CRC** |
| --- | --- | --- |
| **CCL20** | 12.24 (0.001) | 12.24 (0.02) |
| **CXCL1** | 37.74(0.001) | 42.24 (0.001) |
| **CXCL2** | 11.14 (0.006) | 16.47 (0.001) |
| **CXCL3** | 50.72 (0.001) | 70.11 (0.001) |
| **IL8** | 42.95 (0.001) | 173.47 (0.001) |
| *CCL5* | 0.43 (0.029) | 0.58 (0.226) |
| *CCL19* | 0.21 (0.046) | 0.33 (0.266) |
| *CCL21* | 0.07 (0.042) | 0.35 (0.117) |
| *CCL23* | 0.09 (0.001) | 0.22 (0.001) |
